# Effectiveness of Sports Nutrition Education Based on Self-Determination Theory for Male University Rowing Athletes: A Randomized Controlled Trial

**DOI:** 10.3390/nu16060799

**Published:** 2024-03-11

**Authors:** Naoko Enatsu, Jun Seino, Taishi Tsuji, Mitsugi Ogata

**Affiliations:** 1 Japan Institute of Sports Sciences, Japan High Performance Sport Center, 3-15-1 Nishigaoka, Kita-ku, Tokyo 115-0056, Japan; naoko.enatsu@jpnsport.go.jp; 2 Department of Nutrition, Faculty of Medical and Health Sciences, Kiryu University, 606-7 Azami, Kasakake-machi, Midori 379-2311, Gunnma, Japan; 3 Institute of Health and Sport Sciences, University of Tsukuba, 1-1-1 Tennodai, Tsukuba 305-8577, Ibaraki, Japan; tsuji.taishi.gn@u.tsukuba.ac.jp (T.T.); ogata.mitsugi.kb@u.tsukuba.ac.jp (M.O.)

**Keywords:** sports nutrition knowledge, brief self-administered diet history questionnaire, intervention study, nutrition education, male athletes

## Abstract

To resolve problems in the dietary life of university athletes, education is essential to enable athletes to change their own dietary behavior. The purpose of this research was to verify the effectiveness of sports nutrition education based on self-determination theory (SDT). The participants were 36 male university rowers. A stratified randomized comparison test was conducted by student year (SDT group and control group). Sports nutrition education was held three times, via an Internet conferencing system. Furthermore, group work over social media was used for the SDT group. Four evaluations were carried out based on anthropometric measurements, a brief self-administered diet history questionnaire (BDHQ), sports nutrition knowledge test (SNK), and treatment self-regulation questionnaire (TSRQ). The results showed no differences between the two groups. However, for the intragroup factor, “Protein”, a significant difference was evident in the self-determination theory group (50.0 ± 28.5, 78.6 ± 28.1, 81.0 ± 21.5, *p* < 0.000, units: %) and improved knowledge (*p* = 0.002, *p* = 0.002). And for the BDHQ, the self-determination theory group also showed significant differences and increased their intake of green and yellow vegetables, fruits, and dairy products (159.1 ± 74.2–126.7 ± 70.6, *p* = 0.009, 306.0 ± 196.2–195.2 ± 146.1, *p* = 0.020, 257.0 ± 147.0–183.3 ± 167.9, *p* = 0.040, units: g). In conclusion, sports nutrition education based on SDT improved dietary knowledge and increased food requirements for athletes.

## 1. Introduction

### 1.1. Background

Rowing is a competitive sport that requires both anaerobic and aerobic capacity. Although a race lasts only 6–8 min, daily practice sessions are long [1]. In addition, there are lightweight and heavyweight categories, which require different body masses and compositions, requiring individualized nutritional strategies and plans. Additionally, Michael et al. reported that there is an association between patterns of achievement-motivated behavior and performance and future success in rowing [2]. Karen et al. suggested that a well-planned nutrition strategy that includes the careful timing and selection of appropriate foods and fluids helps to maximize training adaptations and, thus, should be an integral part of the athlete’s training program. And they also tell us that food choice is influenced by physiological, social, psychological, and economic factors and varies both within and between individuals and populations, and they say that research is needed to investigate the motivations for athletes’ food choices [3].

Hamaguchi et al. reported that university students often eat takeaway foods or eat outside, and thus have a low awareness of balancing meals and cooking for themselves. Therefore, they pointed out that to raise awareness among university students, practical hands-on guidance is important in order for them to concretely understand and become aware of problematic areas in various directions by learning about problems related to food on their own and understanding their own dietary situation [4]. In addition, Akamatsu suggested that education on eating behavior is an important aspect of nutrition education that incorporates a perspective on eating behavior [5]. Jagim et al. reported a large discrepancy between the perceived amount of energy and nutritional intake and the actual intake among university athletes [6]. These findings highlight the importance of sports nutrition education for university students.

Concerning nutrition education, Contento suggested that to facilitate changes in eating behavior, it is necessary to properly address various factors influencing or determining food choice and eating behavior and to use programs based on educational strategies, learned experience, and evidence [7]. In addition, a nutrition intervention protocol that incorporates a behavioral change model to boost motivation for the 5As (Ask, Advise, Assess, Assist, and Arrange) and motivational interviewing (MI) has been tested for adolescent athletes and has been reported to potentially promote changes in dietary behavior and inform nutrition strategies [8]. This 5A behavior change model, which has also been tested in smoking cessation [9] and weight loss counseling [10], has been reported as an effective tool for behavioral counseling [11]. MI is a client-centered approach developed by Miller [12] that focuses on assisting participants in planning and achieving autonomy to lead healthier and fulfilling lives. Regarding this motivation, it has been reported that Olympic medalists in judo “became intrinsically motivated by developing an interest in diet through a recognition that diet and athletic performance are linked” [13]. Therefore, research on nutrition education for athletes focusing on motivation has attracted attention in a broad range of sports. The formation of athletes’ eating behaviors and assessment of dietary intake is very important because an appropriate dietary intake improves athletes’ health and sports performance [14]. However, few studies have investigated deeper associations. Therefore, we focused on nutrition education research for athletes, focusing on motivation.

This study examines this motivation in terms of intrinsic and extrinsic motivation and focuses on Ryan and Deci’s self-determination theory (SDT), which explains human behavior from the perspective of autonomy [15]. They said that intrinsically motivated behaviors are experienced as being volitional and emanating from one’s self, a point made early on. And in contrast, they also said that extrinsically motivated behaviors occur because of externally imposed reward or punishment [15].

One of its sub-theories, the Basic Psychological Needs Theory, involves three basic psychological needs—autonomy, competence, and relatedness—that lead to intrinsic motivation. This theory is employed in a wide range of areas, including health management and diet therapy, smoking cessation, dentistry, sports, physical activities, physical education, and work and organization.

Contento asserted that nutrition education helps students achieve motivation, self-control, and self-determination when it provides active feedback for the experience of autonomy (Autonomy) and situations where students feel responsible for acting with confidence (Competence) and provides support in the educator–student relationship (Relatedness) [7]. Markland et al. also suggested adopting the SDT perspective to deepen our understanding of the psychological processes involved in MI [16]. Furthermore, Patrick and Williams suggested that SDT and MI, although developed for different purposes and areas, have a great deal of conceptual overlap [17]. An SDT study on athletes reported that autonomy-supportive coaching strengthened the motivational orientation of behavior in high school and college athletes [18]. It has been reported that an SDT-informed intervention for young endurance athletes significantly increased knowledge but did not lead to changes in food intake [19]. In addition, motivation underlies feeding regulation, and the satisfaction and inhibition of basic psychological needs in SDT show how disordered eating occurs and how a person can optimally regulate ongoing eating patterns [20]. Leblanc et al. suggest that nutrition education utilizing self-determination theory appears to be particularly compatible with male individuals [21]. The results of a meta-analysis by Ntoumanis et al. indicate that SDT-based interventions have a positive impact on health parameters [22]. However, the examples of this type of intervention study focusing on male athletes are scarce. And Simona et al. reported that research on the effectiveness of nutrition education and behavior change interventions in athletes is lacking; therefore, they report that additional studies of sufficient rigor (i.e., randomized controlled trials) are needed to demonstrate the benefits of nutrition counseling in athletes [23].

### 1.2. Purpose

Against this background, we speculated that sports nutrition education, by incorporating SDT and creating opportunities to understand an individual’s own diet, might help students improve their food knowledge and improve and sustain their autonomous eating behavior. Therefore, the purpose of this study was to implement an SDT-informed sports nutrition education intervention and identify its effectiveness in inducing autonomous eating behaviors among male university athletes.

## 2. Materials and Methods

### 2.1. Experimental Design

The intervention period was six months, from 13 June 2021 to 27 November 2021. This study comprised a two-group randomized controlled trial.

The groups were blocked, randomized, and assigned according to grade level after the pre-survey. Individuals were assigned code names by the author, and assigned to the intervention group (SDT group) or the control group (hereafter referred to as the COT group). It used the RAND function in Excel to generate a random number and sort the members into two groups. The power calculations were based on the results from a prior study on nutrition knowledge [24]. In this study, the mean difference in knowledge between athletes and coaches was 8 ± 9%, with athletes correct 73 ± 9% and coaches correct 81 ± 9%. Thus, we estimated that the change in knowledge scores that would be achievable and beneficial for the participants was about 8%. (α = 0.05, desired power = 0.80). Based on this, in this study, we have just confirmed G-power; we calculated that there should be 17 athletes in each group, with an expected dropout of 10%. Therefore, we estimated that we needed at least 38 athletes.

### 2.2. Participants

The participants were defined as members of the University of Rowing Club in April 2021 who were training continuously, and excluded were those who had left the club, taken a leave of absence, or switched to being coxswains. Thirty-six male athletes and four staff members, including a coach, a coxswain, a new trainer coach, and a manager, participated in the survey. The male athlete members were assigned to two groups, with 18 members in the SDT group and 18 in the COT group.

**Figure 1:**
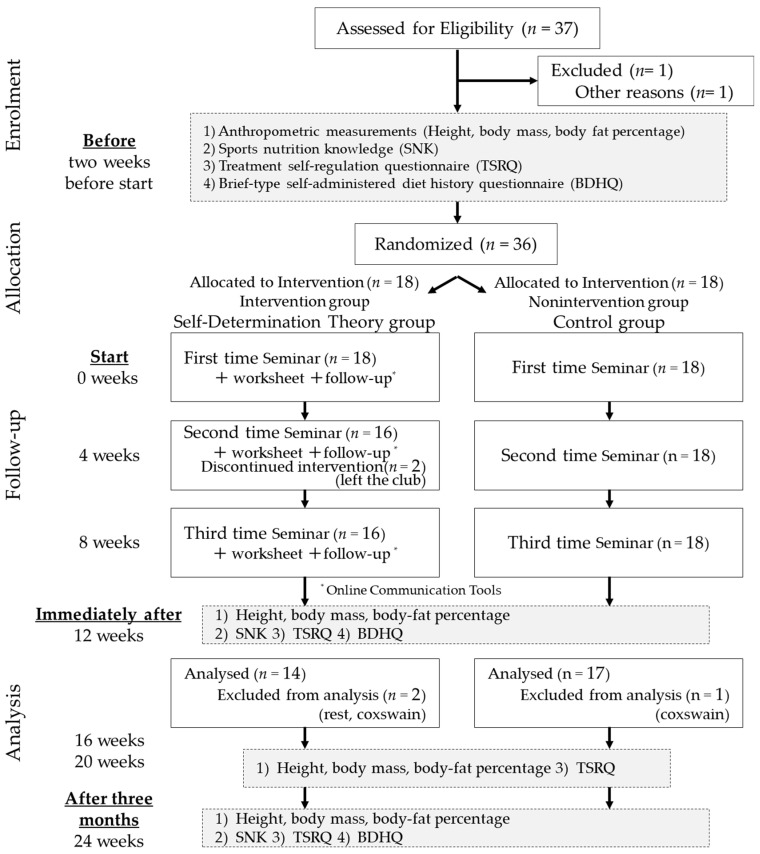
Research design.

An explanatory meeting was held for the participants two weeks before the study program began. An explanation of the study and a consent form were distributed to the participants, who provided free and voluntary informed consent. For the consent of minors, we mailed an explanation of the study, together with a consent form, to their parents or guardians and asked them to give their consent by returning the completed forms to us in a self-addressed envelope. The participants were informed that they would not know which group they would be assigned to at the time of the briefing session. A preliminary survey was conducted on 30 May, two weeks before the program began. The overall study design is shown in Figure 1.

### 2.3. Intervention Program

#### 2.3.1. Program Overview

This study created an intervention program centered on SDT that incorporated the elements of the 5As and MI. The aim of the SDT program was to enhance autonomy, leading to a change in intrinsic behavior, and it included the following elements: (1) Desire for Autonomy, which is a desire for the self-determination of one’s own experiences and actions, and a sense of self-determination of one’s own actions; (2) Desire for Competence, which is a desire to effectively demonstrate one’s ability and competence, and the feeling of being able to demonstrate one’s abilities and talents; and (3) Desire for Relationships (relations and interactions), which is the desire to form good relatedness with others, to be cared for by significant others, and to contribute something for the benefit of others [15]. For the intervention program that incorporated elements of the 5As and MI, we also incorporated elements of the nutrition intervention protocol validated by Lee and Lim [8].

In this study, group sports nutrition education seminars were held once every four weeks for a total of three sessions, using a web-conferencing system. Table S1 presents the overall flow of the program. The program time was 90 min for the SDT group and 60 min for the COT group. After the sports nutrition education seminar, information was shared once a week in small groups (four or five participants), including one manager for both groups, using an online communication tool. The content of the program was decided by two university faculty members specializing in physical education, sports management, coaching, and sports nutrition. One of the authors, a sports nutritionist, provided the guidance.

#### 2.3.2. Sports Nutrition Education Seminars

Educational content and handouts were similar for both the SDT and COT groups, with the SDT group receiving an additional worksheet that included work on Autonomy, Competence, and Relatedness.

The common educational content for both groups was original and based on the content of the nutrition education session adopted by Lee and Lim [8]. In the first month, the Basic Nutrition Concept program included (1) the need for sports nutrition and how to use nutritional information, (2) nutrition and training, (3) regular eating habits, (4) body image, and (5) knowledge of sports nutrition (protein, carbohydrates, and water supplementation). In the second month, the Basic Food Skills program included (1) knowledge of sports nutrition (including supplements), (2) grocery shopping (ingredients and food selection), (3) cooking methods and food hygiene, (4) meal planning, and (5) eating out. In the third month, the Performance Enhancement program consisted of (1) planning light meals (recovery from fatigue and muscle pain), (2) meals before practice and games, (3) meals during practice and games, (4) meals after practice and games, (5) meals on the move, and (6) meals for various purposes (weight gain, weight loss, and weight maintenance). All participants were encouraged to participate in interactive communication through a web conferencing system.

The additional work performed by the SDT group was designed to promote autonomy based on (1) providing rational motivation for the participant activities, (2) understanding the participants’ feelings, and (3) giving the participants a choice of activities [25]. The participants then undertook question-and-answer sessions and group discussions, and used a worksheet that allowed them to discover problems and issues with their own food profiles, their usual cooking, and light meals before and after practice and games. To enhance autonomy, support was provided by clearly communicating expectations and clarifying the process of achievement [26]. Concrete suggestions for improvement were provided before the worksheet was filled out. We ensured that this led to their usual eating habits by asking them to write down, in their own words, the areas for improvement and by actually taking action. The worksheets were collected after the sports nutrition education seminar and returned after checking their content. The checked content was used to send advice via e-mails. Sports, nutrition, and education seminars were held in the evenings after practice, and the participants attended seminars at the boatyard and from their homes.

#### 2.3.3. Group Support Using Online Communication Tools

For the online communication tool, both groups shared one day’s worth of photographs of their meals once a week and were able to consult with the author regarding any questions they had.

In addition, for the SDT group, progress in the tasks was reported within the group based on worksheets completed at the monthly sports nutrition education seminars, allowing the groups to advise each other. The SDT group managers reported their progress to the authors each week, and the authors sent 12 advice e-mails to each group within 2–3 days of the report. This program was designed to link sports nutrition education with group support using online communication tools.

### 2.4. Survey and Measurement Instruments

The survey/measurement items included anthropometric measurements (height, weight, and body fat percentage), a brief self-administered diet history questionnaire (BDHQ), a sports nutrition knowledge questionnaire (SNK), and a treatment self-regulation questionnaire on the self-regulation of eating habits (TSRQ). The primary outcome comprised anthropometric measurements and the BDHQ; the secondary outcome comprised the SNK and TSRQ.

Anthropometric measurements were taken using equipment that estimates body composition using bioelectrical impedance analysis (BIA). The following seven points were standardized when measuring body mass and body fat percentage: (1) measurements were taken two hours after eating; (2) urination and defecation were completed before measurement; (3) measurements immediately after exercise were avoided; (4) measurements in the presence of dehydration or swelling were avoided; (5) measurements at low temperatures or during hypothermia were avoided; (6) measurements at high temperatures were avoided; and (7) measurements immediately after bathing were avoided. Anthropometric measurements were taken with a manager who was thoroughly familiar with this method.

Kobayashi et al. [27] developed the BDHQ, which retains the features of the self-administered dietary history questionnaire developed by Sasaki et al. [28] by simplifying its structure, responses, and data processing. We used the BDHQ to survey nutrient and other intakes in the previous month. The manager distributed the BDHQs and collected them within three days. The manager and authors checked for omissions and errors on the day of collection; if recompletion was required, the manager requested correction within a few days and the resulting responses were collected once more.

The SNK was based on a questionnaire (40 questions) developed by Walsh et al. [29] and surveyed food knowledge, categorized into five sections: (1) Training Schedule and Positioning with Training, (2) Eating and Hydration Habits and Awareness, (3) Attitudes Toward Nutrition Intake, (4) Nutrition Knowledge, and (5) Nutrition Information Sources and Nutrition Education that Might be Needed in the Future.

The TSRQ was developed by Ryan and Connell [30] to assess autonomous self-regulation, and was first used for health purposes by Williams et al. [31]. It is now widely used in behavioral change studies in medical settings. Subsequently, Levesque et al. validated the utility of healthy behaviors [32]. The TSRQ consists of a series of questions about why people engage in or attempt to engage in healthy behaviors, undergo treatment for illness, change unhealthy behaviors, follow a treatment plan, or engage in other health-related behaviors (Center for Self-Determination Theory online) [33]. There are four types of TSRQ: smoking cessation, dietary modifications, regular exercise, and responsible drinking. In this study, we used a modified version of the diet. Furthermore, respondents were asked to respond to the questions by viewing their health as “health on the playing field”. The TSRQ was used after we reconfirmed its contents, which had been translated by a company specializing in translating research papers.

These questionnaires have been developed and validated [27,29,34].

These surveys and measurements were conducted two weeks before the start of the program (before), immediately after the program’s conclusion (immediately after), and three months after the program’s conclusion (after three months). During the program’s implementation period, the participants’ body composition in terms of height, weight, and body fat percentage was measured every four weeks, and anthropometric measurements and diet self-control were conducted every four weeks as a continuing evaluation for three months after the program ended.

### 2.5. Statistical Analysis Methods

Normality assumption was confirmed using the Shapiro–Wilk test. The program was subjected to parametric tests before it started, immediately afterward, and three months later. Anthropometric data were obtained as mean and standard deviation.

In this study, the nutrient intake of ten items and the nutrient intake by food group of fifteen items were used in the BDHQ. The nutrients selected were “iron, vitamin D, calcium, and the antioxidant vitamin C”, which are reported to be particularly important for athletes [35]. In addition, vitamin B1 and dietary fiber, which are easily deficient in the university’s rowing club, were selected.

The 14 SNK questions were divided into five items (Total Knowledge Score, Energy and Replenishment, Hydration, Supplements, and Protein), and the percentages of correct answers were analyzed for each item.

The TSRQ of 15 questions was divided into four items. The means of the responses to each question were calculated for three items: autonomous motivation, externally controlled motivation, and non-motivation. The fourth item, the Relative Autonomous Motivation Index, is calculated by subtracting the mean value of externally controlled reasons from that of autonomous reasons.

The results of the BDHQ, SNK, and TSRQ were expressed as means and standard deviations. Unpaired *t*-tests were used for pre-program group comparisons. The Levene test was checked before the ANOVA to confirm equal variances. Repeated two-way analysis of variance was used for between-group and within-group factors, and the Bonferroni method was used for multiple comparison tests when significant differences were confirmed. The statistical significance level was set at 5%. Statistical analysis software was IBM^®^ SPSS Statistics 28.0 for Windows.

### 2.6. Ethical Considerations

The University of Tsukuba Research Ethics Committee reviewed the ethical considerations for conducting this study in accordance with the Declaration of Helsinki and approved it on 20 May 2021 (issue no. Tai 020-176) [36].

## 3. Results

### 3.1. Participant Flow and Number of Participants Analyzed

Two members left the club, and there was one leave of absence and two coxswain transfers, resulting in a total of 31 participants for analysis: 14 in the SDT group and 17 in the COT group.

### 3.2. Differences between Groups before the Program

The results of the anthropometric measurements, SNK, and TSRQ are listed in Table S2. No significant differences were observed in any parameters between the SDT and COT groups. The BDHQ results are shown in Table S3. Before, The SDT and COT groups had no significant differences in nutrient intake, but there were significant differences in meat (*p* = 0.043) and fat and oil (*p* = 0.004) intake by food group.

### 3.3. Immediately after and after Three Months—Anthropometric Measurements, Sports Nutritional Knowledge Questionnaire (SNK), and Treatment Self-Regulation Questionnaire (TSRQ)

The results of the anthropometric measurements, SNK, and TSRQ are listed in Table S2. No significant differences were observed in any parameters between the SDT and COT groups. Table S4 lists the results of the anthropometric measurements, SNK, and TSRQ. Between the SDT and COT groups, there were no significant differences in body mass, body fat percentage, lean body mass, or lean body mass/m. There were no differences between the SDT and COT groups in intragroup factors for immediately after and before, after three months and before, or after three months and immediately after. There was no significant difference in the total SNK knowledge score between the SDT and COT groups in the between-groups factor. On the other hand, there was no significant difference in the main effect of time on the within-group factor (*p* = 0.004). The SDT group had 78.6 ± 12.5% for before and 87.2 ± 7.5% for immediately after, whereas the COT group had 75.6 ± 10.1% for before and 83.6 ± 8.3% for immediately after. Thereafter, there was a significant difference (*p* = 0.003) between immediately after and before in a subsequent multiple comparison test, with an increase in immediately after compared with before. In the between-group factor for protein, there was no significant difference between the SDT and COT groups. In contrast, there was a significant difference in the main effect of time on the within-group factor (*p* < 0.001).

In the SDT group, the results were 50.0 ± 28.5% for before, 78.6 ± 28.1% for immediately after, and 81.0 ± 21.5% for after three months. In the COT group, the results were 56.9 ± 22.9% for before, 72.5 ± 27.0% for immediately after, and 68.6 ± 24.9% for after three months. In the subsequent multiple comparison tests, there was a significant difference between immediately after and before (*p* = 0.002), and between after three months and before (*p* = 0.002). There were no significant differences between or within the groups in terms of energy, supplementation, hydration, or supplements. There were no significant differences between the SDT and COT groups on the TSRQ autonomous motivation subscale, externally controlled motivation subscale, non-motivation subscale, or the Relative Autonomous Motivation index in terms of between-group and within-group factors.

### 3.4. Immediately after and after Three Months—Brief Dietary History Questionnaire (BDHQ)

The BDHQ results are shown in Table S3. The BDHQ results are presented in Table 1. In terms of between-group factors, there was no significant difference in carbohydrate intake between the SDT and COT groups. However, only the main effect of time was significant (*p* = 0.021), and a subsequent multiple comparison test revealed no significant difference. For the between-group factor, there was no significant difference in calcium between the SDT and COT groups. However, only the main effect of time was statistically significant (*p* = 0.026). Thereafter, subsequent multiple comparison tests showed no significant differences. Moreover, there were no significant differences in energy, protein, fat, iron, vitamin D, vitamin B1, vitamin C, or dietary fiber levels between or within groups.

There were no significant differences in the intake of green and yellow vegetables between the SDT and COT groups in terms of the intake of each food group and the between-group factors. However, the main effect of time on the within-group factors showed a significant difference (*p* = 0.009). The intake of green vegetables in the SDT group was 126.7 ± 70.6 g for before and 159.1 ± 74.2 g for immediately after, whereas in the COT group, it was 99.8 ± 69.2 g for before and 159.9 ± 99.3 g for immediately after. A subsequent multiple comparison test revealed a significant difference (*p* = 0.009) immediately after and before. Fruits were not significantly different between the SDT and COT groups in terms of between-group factors. However, there was a significant difference in the main effect of time on within-group factors (*p* = 0.013).

In the SDT group, the intake of fruits was 195.2 ± 146.1 g for before and 306.0 ± 196.2 g for immediately after, whereas in the COT group, it was 133.2 ± 152.4 g for before and 215.3 ± 208.6 g for immediately after. Thereafter, multiple comparison tests showed a significant difference (*p* = 0.020) immediately after and before the intervention.

There were no significant differences in the between-group factors for dairy product production between the SDT and COT groups. However, there was a significant difference in the main effect of time on within-group factors (*p* = 0.012). The intake of dairy products was 183.3 ± 167.9 g for before and 257.0 ± 147.0 g for immediately after in the SDT group, and 177.3 ± 93.3 g for before and 262.6 ± 134.4 g for immediately after in the COT group. A subsequent multiple comparison test revealed a significant difference (*p* = 0.040) immediately after and before.

There were no significant differences between or within the groups for cereal grains, potatoes, sugar and sweeteners, pulses, other vegetables, fish and shellfish, meats, eggs, fats and oils, confectioneries, beverages, or seasonings and spices.

## 4. Discussion

This study implemented SDT-informed sports nutrition education among a male university rowing club with the goal of not only improving sports nutrition knowledge but also establishing an autonomous dietary environment. The results showed no differences between the two groups in terms of the anthropometric measurements, BDHQ, SNK, and TSRQ. Thus, the effectiveness of education has not yet been demonstrated. However, anthropometric measurements included the summer months, when appetites are more likely to decrease, but weight and lean body mass did not decrease significantly. In an intervention program combining nutrition education and food environment intervention in a randomized controlled trial similar to this study, the authors reported that the main outcome, the BDHQ survey, confirmed changes in intake by food group [37]. In our study, too, for the BDHQ results for intake by food group, from the time immediately before the intervention to the time immediately after it, the green and yellow vegetable, fruit, and dairy product intake increased for both groups. In terms of food knowledge, the aggregate knowledge score from the SNK and the scores for protein items improved immediately after the intervention compared with those before the intervention and, with respect to protein items alone, remained higher for three months. However, the results indicated no significant differences between the two groups.

For intrinsic motivation, internalization, where a person integrates extrinsically motivated behaviors as their own, plays an important role, which requires the basic psychological needs of Autonomy, Competence, or Relatedness to be fulfilled [15]. Roth et al. [38] argued that autonomous motivation for learning is promoted by expanding support for autonomy. Based on these reports, in this study, support was provided to the SDT group so that the participants used a worksheet in the sports nutrition education seminars to review their everyday meals, write down areas for improvement in their own words, and link these exercises to actual actions. We attempted to strengthen this mechanism by providing group support using online communication tools. However, our results do not support the effectiveness of this mechanism. According to Murray et al., the factors that contribute to problematic cooking practices among university students include insufficient knowledge and skills in cooking, financial insecurity, inadequate information on healthy eating, and time and lifestyle constraints. Therefore, it is important to design an effective and strategic program to promote motivation [39]. In the case of university rowing clubs, some clubs provide meals in their dormitories, but if the environment is not conducive, they often cook their own meals. Therefore, specific suggestions were needed to create a food environment for university rowers. In this study, food choices and cooking methods were proposed to improve cooking skills at home. This might explain why the effects were observed in both groups immediately after the intervention, leading to changes in eating behavior. Heikkilä et al. [19] reported, in a study on SDT-informed nutrition education intervention, that undergoing the education three times significantly increased nutrition knowledge, but did not lead to changes in food intake. However, in the current study, the participants in both groups established learning goals in a sports nutrition education seminar. This led not only to improved nutritional knowledge, but also to an increase in food intake. The SDT group was asked to look at pictures of their own food, examine “the items that I am missing” and set goals so that they would be intrinsically motivated. Thus, the program was unique, in that it was designed to use learning goals to make students “aware”. The approach based on the 5As and MI, which encouraged food selection, was effective, as we believe that it brought about such behaviors and practices in the SDT group.

All seminars were conducted using synchronous distance education and a web conferencing system. As this learning format was entirely new to the participants, we believe that it played a role in making it more difficult for the variances between the groups to manifest. In contrast, Liyun et al. reported that for knowledge acquisition, synchronous distance education was not significantly different from conventional education in terms of learning effectiveness, and the level of satisfaction was also high [40]. The adoption of synchronous distance education in this study led not only to knowledge, but also to action and practice in both groups. We can infer from this that the results demonstrate the effectiveness of synchronous distance education, regardless of the SDT-based approach.

Because intrinsic framing of goals produces effects such as deeper engagement in learning activities, better conceptual learning, and higher persistence in learning activities [41] and learning goals, in which individuals seek to increase their competence, promote challenge-seeking behaviors and a mastery-orientated response to failure [42], the intrinsic framing of goals is likely to contribute to the subsequent continuation of specific habits. Therefore, considering the need for practical hands-on guidance, as discussed by Hamaguchi et al. [4], providing face-to-face nutrition education involving cooking practice and food tasting still leads to the creation of distinctive eating experiences: feeling the taste of food, thinking about how to achieve that taste, and discovering and being impressed by great food. This, in turn, might lead to the continuation of such experiences. In order to implement effective sports nutrition education, we would like to construct an ongoing program that can maintain the intrinsic motivation of athletes.

This study is limited in a number of dimensions. In this study, we focused on feasibility studies [43] and we designed the study to fully consider acceptability, demand, implementation, and practicality, but we believe that we did not fully consider adaptation, integration, extension, and the limited efficacy evaluation. This is because it was a randomized controlled trial administered within a single university rowing club by grade, and we needed to guarantee the quality of the content and make it uniform to a certain extent between the two groups. Nutrition education had to be provided without significantly varying it between different participants, and the manager and all other staff members had to treat all participants similarly. In addition, because they shared the same practice environment and ate in the same place, they inevitably interacted with one another and influenced each other. Within the club, athletes were mutually supportive, and the team’s group cohesiveness was high. The design of a controlled trial, “creating a COT group that does nothing”, might, therefore, have limitations in sports practice. In this study, the research design was developed without eliminating existing positive team practices. As Tanaka and Shigematsu [44] state, “rather than creating a control group that is not allowed to do anything, we should focus on participant front of us”, and we believe that researchers should pay attention to their research participants and have flexible thinking and broad perspectives. We also used expert-translated questionnaires for the SNK and TSRQ, which have not been validated in our country and need to be validated. As for the BDHQ, it is a questionnaire that has been validated in our country, but it has several issues. Since the amount of food is not specified and the method is to select the frequency, the difference may be large if the amount of food per meal differs [27]. Regarding food weight, rice and cereal grains include water from cooking, and tofu and soy milk, which are legumes, also include water. And in Japan, rice is often eaten as polished rice and fruits are peeled, so the fiber content may be low relative to the food weight. Other limitations are that the university students in this team do not have the habit of drinking cola and other sugar-containing beverages when actually surveyed about their eating habits, probably due to team rules and culture, so the amount of beverage-derived carbohydrates is not so high. However, as reported by Androniki, it is undeniable that the dietary questionnaire may have underestimated the number of subjects, and some sources of error still remain [45]. Therefore, in this study, as in the numerous previous studies that have been identified, the fact of underestimation is undeniable, and we suggest that it is a limitation of the study and an issue for future research. Finally, at the time of the survey, due to the COVID-19 pandemic, meals could not be provided within the team. To address this issue, both groups received assistance with meals immediately after the outbreak through an “emergency special donation” from the team alumni. We believe that this potentially had an uncontrollable influence on the variance between the SDT and COT groups.

## 5. Conclusions

The results of this study demonstrate that SDT-informed sports nutrition education, through improvement in nutrition knowledge, can potentially lead to the practice and continuation of improved eating behaviors. These effects were clearly demonstrated by the actual food intake. Therefore, body mass, one indicator of athlete health, was maintained, and lean body mass was not significantly reduced. Additionally, the provision of support designed to promote autonomy allowed us to discover that autonomy potentially facilitates intrinsic motivation. In developing a program utilizing SDT in a department that is student-driven, the cooperation of the student staff may lead to continued food awareness and eating behavior, which may affect the overall team’s bottom line. The program used in this study could potentially promote sports nutrition education.

## Figures and Tables

**Table 1:** Results of between-group and within-group factor analysis for the BDHQ.

		Grouping Factor	Within-Group Factor				
		Reciprocal Action Group × Time *p* ^†^	Time Main Effects *p* ^†^	Immediately After–Before *p* ^‡^	After Three Months–Before *p* ^‡^	After Three Months–Immediately after *p* ^‡^	Multiple Comparison Test
**BDHQ (Nutrient intake)**							
	Energy (kcal)	0.413	0.059				
	Protein (g)	0.652	0.114				
	Fat (g)	0.853	0.810				
	Carbohydrate (g)	0.307	0.021 *	0.059	0.090	1.000	n.s
	Calcium (mg)	0.939	0.026 *	0.054	0.178	1.000	n.s
	Iron (mg)	0.757	0.056				
	Vitamin D (μg)	0.314	0.059				
	Vitamin B_1_ (mg)	0.832	0.080				
	Vitamin C (mg)	0.854	0.050				
	Dietary fiber (g)	0.967	0.295				
**BDHQ (Intake by food group)**							
	Cereal grains (g)	0.305	0.068				
	Potatoes (g)	0.806	0.553				
	Sugar and sweeteners (g)	0.864	0.051				
	Pulses (g)	0.407	0.121				
	Green and yellow vegetables (g)	0.450	0.009 *	0.009 *	0.204	0.735	Before < Immediately After
	Other vegetables (g)	0.769	0.435				
	Fruits (g)	0.735	0.013 *	0.020 *	0.619	0.267	Before < Immediately After
	Fish and shellfish (g)	0.467	0.070				
	Meats (g)	0.521	0.527				
	Eggs (g)	0.973	0.871				
	Dairy products (g)	0.657	0.012 *	0.040 *	0.770	0.076	Before < Immediately After
	Fats and oils (g)	0.252	0.322				
	Confectioneries(g)	0.688	0.418				
	Beverages (g)	0.713	0.052				
	Seasonings and spices (g)	0.394	0.148				

* *p* < 0.05: Significant difference. n.s.: Not significant. ^†^ A two-way ANOVA was conducted on the between-group and within-group factors. ^‡^ Multiple comparison tests using the Bonferroni method were performed for before, after, and after three months for within-group factors. < less than. BDHQ: brief self-administered diet history questionnaire.

## Data Availability

Data are contained within the article and Supplementary Materials.

## Supplementary Materials

The following supporting information can be downloaded at: https://www.mdpi.com/article/10.3390/nu16060799/s1, Table S1: Program overview; Table S2: Between-group differences in anthropometric measurements, the SNK and the TSRQ (prior); Table S3: Between-group differences in the BDHQ (prior); Table S4: Results of between-group and within-group factor analyses of anthropometrics, SNK, and TSRQ.
