# Supplementary material for: Effectiveness of Sports Nutrition Education Based on Self-Determination Theory for Male University Rowing Athletes: A Randomized Controlled Trial
Source: Nutrients. 2024 Mar 11;16(6):799. doi: 10.3390/nu16060799 (PMC10975097; doi:10.3390/nu16060799)
Supplement: Supplementary file 1 [file nutrients-16-00799-s001.zip › nutrients-2878750-supplementary.pdf]

Supplementary Material

Table S1. Program Overview.

			Before	Implementation period												Immediately after	Continued Evaluation		
		MONTH		1st				2nd				3rd					4th	5th	6th
		DAY	5/30	6/13	6/20	6/27	7/4	7/10<br>7/11	7/18	7/25	8/1	8/7<br>8/8	8/15	8/22	8/29	9/4	10/2	10/30	11/27
		WEEK	–2	0	1	2	3	4	5	6	7	8	9	10	11	12	16	20	24
**Program Flow**	SDT group	Seminar（60 minutes）		✔				✔				✔							
		+ worksheet（30 minutes）		✔				✔				✔							
		Share meal photos and your issues（Online Communication Tools）		✔	✔	✔	✔	✔	✔	✔	✔	✔	✔	✔	✔	✔			
		+ follow-up (mail)		✔	✔	✔	✔	✔	✔	✔	✔	✔	✔	✔	✔	✔			
	COT group	Seminar（60 minutes）		✔				✔				✔							
		Free, contact dietitian if you have questions（Online Communication Tools）		✔	✔	✔	✔	✔	✔	✔	✔	✔	✔	✔	✔	✔			
**Survey and measurement**		Research Description and Consent Form	✔																
	1	Anthropometric measurements (Height, weight, body fat percentage)	✔			✔				✔				✔		✔	✔	✔	✔
	2	Sports Nutrition Knowledge (SNK)	✔													✔			✔
	3	Treatment self-regulation questionnaire (TSRQ)	✔													✔	✔	✔	✔
	4	Brief-type self-administered diet history questionnaire (BDHQ)	✔													✔			✔

SDT group: Self-Determination Theory group　COT group: Control group

Table S2. Between-group differences in anthropometric measurements, SNK and TSRQ.

	Self-Determination Theory group (*n* = 14)			Control group (*n* = 17)			p value†
	Before	Immediately after	After three months	Before	Immediately after	After three months	
	mean value±SD	mean value±SD	mean value±SD	mean value±SD	mean value±SD	mean value±SD	
**Anthropometric measurements**							
Age (yrs)	19.6 ± 1.5	19.9 ± 1.6	19.9 ± 1.6	19.6 ± 1.3	19.9 ± 1.5	19.9 ± 1.5	0.973
Height (cm)	173.9 ± 4.8	173.9 ± 4.8	173.9 ± 4.9	173.1 ± 5.6	172.9 ± 5.5	172.8 ± 5.7	0.682
Weight (kg)	72.0 ± 7.9	71.5 ± 6.2	72.4 ± 6.0	68.3 ± 6.2	69.2 ± 6.3	69.6 ± 5.4	0.152
Body fat (%)	14.6 ± 4.1	14.3 ± 3.9	14.4 ± 3.0	14.0 ± 4.3	12.7 ± 3.9	13.3 ± 3.4	0.712
Lean body mass (kg)	61.4 ± 5.7	61.2 ± 5.6	62.0 ± 5.6	58.8 ± 7.1	60.4 ± 5.8	60.3 ± 4.8	0.290
Lean body mass/m (kg)	35.3 ± 2.8	35.2 ± 2.7	35.6 ± 2.5	33.9 ± 3.4	34.9 ± 2.5	34.8 ± 2.0	0.251
**SNK**							
Total SNK score (%)	78.6 ± 12.5	87.2 ± 7.5	87.2 ± 8.0	75.6 ± 10.1	83.6 ± 8.3	79.0 ± 15.9	0.475
Energy and refueling (%)	84.5 ± 12.2	90.5 ± 12.6	91.7 ± 10.8	77.5 ± 19.5	86.3 ± 14.7	81.4 ± 17.6	0.228
Hydration (%)	100.0 ± 0.0	100.0 ± 0.0	100.0 ± 0.0	100.0 ± 0.0	100.0 ± 0.0	94.1 ± 16.6	—
Supplements (%)	81.0 ± 25.2	81.0 ± 17.1	76.2 ± 15.6	74.5 ± 30.1	78.4 ± 23.4	74.5 ± 25.1	0.529
Protein (%)	50.0 ± 28.5	78.6 ± 28.1	81.0 ± 21.5	56.9 ± 22.9	72.5 ± 27.0	68.6 ± 24.9	0.463
**TSRQ**							
Autonomous motivation subscale	5.05 ± 0.96	5.48 ± 0.93	5.44 ± 1.09	5.29 ± 1.34	5.49 ± 0.99	5.40 ± 1.06	0.568
Externally controlled motivation subscale	3.07 ± 1.07	2.81 ± 1.12	3.27 ± 1.34	2.92 ± 1.12	3.23 ± 0.92	3.70 ± 1.34	0.708
Nonmotivation subscale	2.74 ± 1.10	2.67 ± 0.93	3.14 ± 1.25	3.08 ± 1.48	3.18 ± 1.22	3.12 ± 1.41	0.482
Relative Autonomous Motivation Index	1.98 ± 1.62	2.67 ± 1.64	2.17 ± 1.93	2.37 ± 1.53	2.26 ± 1.19	1.71 ± 1.89	0.490

† No paired t-test was performed for differences between groups before the start of the study.

SD: Standard Deviation

SNK: Sports Nutrition Knowledge

TSRQ: Treatment Self-Regulation Questionnaire

Table S3. Between-group differences in BDHQ.

	Self-Determination Theory group（n = 14）			Control group（n = 17）			
	Before	Immediately after	After three months	Before	Immediately after	After three months	p value†
	mean value±SD	mean value±SD	mean value±SD	mean value±SD	mean value±SD	mean value±SD	
**BDHQ (Nutrient intake)**							
Energy (kcal)	2764 ± 912	3224 ± 541	3295 ± 713	3000 ± 679	3118 ± 756	3165 ± 919	0.416
Protein (g)	83.5 ± 32.9	95.8 ± 24.8	97.6 ± 28.7	94.1 ± 27.2	95.9 ± 26.3	104.2 ± 34.6	0.334
Fat (g)	63.5 ± 22.3	68.2 ± 20.0	69.0 ± 19.8	75.5 ± 21.3	76.7 ± 27.6	75.5 ± 34.5	0.137
Carbohydrate (g)	448.5 ± 148.8	539.8 ± 93.3	551.2 ± 118.5	468.3 ± 124.4	494.7 ± 117.4	499.7 ± 132.4	0.690
Calcium (mg)	528.7 ± 325.0	633.7 ± 251.7	601.2 ± 296.5	523.6 ± 161.2	659.6 ± 233.0	615.8 ± 185.2	0.958
Iron (mg)	8.2 ± 4.0	9.4 ± 2.8	9.3 ± 4.1	8.5 ± 2.1	9.4 ± 3.3	10.1 ± 3.7	0.850
Vitamin D (μg)	8.4 ± 4.4	9.2 ± 6.0	10.2 ± 6.1	7.5 ± 5.2	8.1 ± 4.7	13.3 ± 11.3	0.597
Vitamin B_1 (mg)	0.9 ± 0.4	1.1 ± 0.2	1.1 ± 0.3	1.0 ± 0.3	1.1 ± 0.4	1.2 ± 0.4	0.455
Vitamin C (mg)	115.9 ± 54.8	141.7 ± 59.4	132.1 ± 74.0	102.5 ± 47.2	131.8 ± 79.2	131.3 ± 65.4	0.469
Dietary fiber (g)	12.3 ± 5.4	13.6 ± 4.1	13.8 ± 4.9	13.5 ± 3.7	14.4 ± 5.4	14.8 ± 5.3	0.461
**BDHQ（Intake by food group）****							
Cereal grains (g)	955.3 ± 305.6	1099.6 ± 245.1	1193.5 ± 268.2	1020.1 ± 327.9	1022.3 ± 207.5	1074.9 ± 285.0	0.577
Potatoes (g)	25.8 ± 25.1	26.2 ± 38.8	28.9 ± 22.5	31.3 ± 32.8	30.8 ± 32.0	42.5 ± 42.4	0.610
Sugar and sweeteners (g)	2.7 ± 2.5	2.9 ± 2.7	3.6 ± 2.1	2.5 ± 2.0	2.3 ± 1.6	3.4 ± 1.9	0.861
Pulses (g)	67.4 ± 67.1	76.4 ± 72.8	53.0 ± 56.9	46.6 ± 34.2	62.5 ± 42.5	52.5 ± 30.5	0.305
Green and yellow vegetables (g)	126.7 ± 70.6	159.1 ± 74.2	138.0 ± 81.8	99.8 ± 69.2	159.9 ± 99.3	146.6 ± 83.6	0.295
Other vegetables (g)	125.0 ± 75.8	113.5 ± 61.6	137.4 ± 75.5	174.5 ± 95.7	150.2 ± 106.9	163.4 ± 78.2	0.127
Fruits (g)	195.2 ± 146.1	306.0 ± 196.2	221.4 ± 147.8	133.2 ± 152.4	215.3 ± 208.6	180.5 ± 139.9	0.261
Fish and shellfish (g)	48.0 ± 29.5	64.7 ± 41.2	62.9 ± 44.3	37.2 ± 30.8	51.3 ± 38.6	71.2 ± 76.2	0.330
Meats (g)	104.2 ± 46.1	111.8 ± 33.4	122.2 ± 42.9	156.2 ± 86.9	134.5 ± 60.1	153.8 ± 87.9	0.043 *
Eggs (g)	57.6 ± 50.9	60.1 ± 53.6	62.9 ± 50.7	65.8 ± 36.4	65.0 ± 49.3	68.2 ± 41.8	0.606
Dairy products (g)	183.3 ± 167.9	257.0 ± 147.0	219.7 ± 146.4	177.3 ± 93.3	262.6 ± 134.4	184.8 ± 95.7	0.900
Fats and oils (g)	16.4 ± 4.4	15.6 ± 5.5	16.8 ± 6.6	23.7 ± 8.3	20.7 ± 7.8	18.7 ± 12.1	0.004 *
Confectioneries(g)	11.5 ± 11.2	7.4 ± 9.5	14.0 ± 16.1	25.6 ± 52.5	12.8 ± 23.7	18.8 ± 22.5	0.333
Beverages (g)	445.7 ± 289.3	656.5 ± 443.9	529.1 ± 518.1	375.9 ± 318.7	482.4 ± 463.8	382.5 ± 351.1	0.532
Seasonings and spices (g)	174.6 ± 147.9	298.0 ± 191.4	255.6 ± 177.7	268.0 ± 186.7	289.7 ± 150.1	285.9 ± 239.6	0.140

* $p < 0.05$: Significant difference

† No paired t-test was performed for differences between groups before the start of the study.

SD: Standard Deviation

BDHQ: Brief-type self-administered diet history questionnaire

**Foods in each food group

Cereal grains : bread, buckwheat, udon (thick wheat noodles), Chinese noodles, pasta, meshi (cooked rice)

Potatoes : potato(All types)　Sugar and sweeteners : sugars, cooking sugar　Pulses : tofu, fried tofu, natto

Green and yellow vegetables : broccoli, carrots, pumpkin, tomatoes　Other vegetables : lettuce, cabbage, radish, root vegetables

Fruits : oranges, strawberries　Fish and shellfish : fish, squid, octopus, shrimp, shell　Meats : chicken, pork, beef, ham

Eggs : eggs　Dairy products : milk, ice cream　Fats and oils : cooking oil

Confectioneries : western confectionery (cake, biscuits, crackers, snacks, chocolate), Japanese confectionery(rice crackers)

Beverages : green tea, black tea, oolong tea, coffee, cola, Japanese sake, beer shochu, whiskey, wine

Seasonings and spices : salt, mayonnaise, soy sauce, miso soup, noodles soup

Table S4. Results of between-group and within-group factor analyses of anthropometrics, SNK, and TSRQ.

	Grouping factor	Within-group factor				
	Reciprocal action Group × Time $p^\dagger$	Time Main Effects $p^\dagger$	Immediately after — Before $p^\ddagger$	After three months — Before $p^\ddagger$	After three months — Immediately after $p^\ddagger$	Multiple comparison test
**Anthropometric measurements**						
Age (y)	0.993	0.099				
Height (cm)	0.367	0.585				
Weight (kg)	0.296	0.206				
Body fat (%)	0.571	0.323				
Lean body mass (kg)	0.253	0.154				
Lean body mass/m (kg)	0.250	0.151				
**SNK**						
Total SNK score	0.510	0.004*	0.003*	0.078	1.000	Before < Immediately after
Energy and refueling	0.711	0.122				
Hydration	0.183	0.183				
Supplements	0.884	0.698				
Protein	0.200	< 0.001*	0.002*	0.002*	1.000	Before < Immediately after<br>Before < After three months
**TSRQ**						
Autonomous motivation subscale	0.571	0.077				
Externally controlled motivation	0.313	0.058				
Nonmotivation subscale	0.311	0.378				
Relative Autonomous Motivation	0.095	0.057				

* $p < 0.05$: Significant difference

† A two-way ANOVA was conducted on the between-group and within-group factors.

‡ Multiple comparison tests using the Bonferroni method were performed for before, after, after three months for within-group factors.

< less than, > more than

SNK: Sports Nutrition Knowledge

TSRQ: Treatment Self-Regulation Questionnaire
